# First record of the hippolytid shrimp *Hippolyteaustraliensis* (Stimpson, 1860) (Crustacea, Decapoda) from China

**DOI:** 10.3897/BDJ.12.e119510

**Published:** 2024-03-26

**Authors:** Zhibin Gan, Xinzheng Li

**Affiliations:** 1 Institute of Oceanology, Chinese Academy of Sciences, Qingdao, China Institute of Oceanology, Chinese Academy of Sciences Qingdao China; 2 University of Chinese Academy of Sciences, Beijing, China University of Chinese Academy of Sciences Beijing China; 3 Center for Ocean Mega-Science, Chinese Academy of Sciences, Qingdao, China Center for Ocean Mega-Science, Chinese Academy of Sciences Qingdao China; 4 Laboratory for Marine Biology and Biotechnology, Pilot National Laboratory for Marine Science and Technology (Qingdao), Qingdao, China Laboratory for Marine Biology and Biotechnology, Pilot National Laboratory for Marine Science and Technology (Qingdao) Qingdao China

**Keywords:** Hippolytid shrimp, *Hippolyteaustraliensis*, new record, South China Sea

## Abstract

**Background:**

Two specimens of the genus *Hippolyte* were examined from the caridean collections of the Marine Biological Museum, Chinese Academy of Sciences. These specimens were captured in the South China Sea in 1987. Detailed inspection revealed that their morphological features closely match the (re)descriptions of *Hippolyteaustraliensis*, especially in the presence of a long, distinct lateral carina on the rostrum, a dorsally unarmed rostral border, and four prominent terminal spines on the dactylus of the last three pereiopods.

**New information:**

The discovery of *Hippolyteaustraliensis* in Chinese waters represents a significant expansion of its geographic distribution from the South Pacific to the North Pacific. Furthermore, an additional taxonomical report of *H.australiensis* is provided since its retrieval in 2001.

## Introduction

The genus *Hippolyte* Leach, 1814 currently comprises 40 valid species occurring from tropical to temperate waters of worldwide oceans (WoRMS, https://www.marinespecies.org/aphia.php?p=taxdetails&id=106987). In the Indo-West Pacific, more than 14 species were recognised. However, only four species, namely *Hippolytechacei* Gan & X Li, 2019, *H.nanhaiensis* Gan & X Li, 2019, *H.ngi* Gan & X Li, 2017 and *H.ventricosa* H. Milne Edwards, 1837, have been recorded from Chinese waters, that all occurred in the seagrass beds of the South China Sea (Gan and Li 2017a, Gan and Li 2017b, Gan and Li 2019). Amongst these, *H.chacei* is distinct, while the other three species belong to the “*H.ventricosa* H. Milne Edwards, 1837” species complex. This species complex comprises more than nine closely-similar species in terms of morphology, along with over four undescribed species identified through molecular genetics (Gan and Li 2019).

Stimpson (1860) reported *Virbiusaustraliensis*, a junior synonym of *H.australiensis*, from Sydney Harbour, Australia, with a brief original description. Bate (1863) also briefly described *Caradinacincunnuli*, another junior synonym of *H.australiensis*, from Gulf St Vincent, Australia. Kemp (1914) transferred *V.australiensis* to the genus *Hippolyte*. Due to the unspecfic original description, Holthuis (1947) and Chace (1997) both regarded *H.australiensis* as a junior synonym of *H.ventricosa*. In 2001, d’Udekem d’Acoz provided a detailed re-description of *H.australiensis*, based on the syntypes of *C.cincunnuli* and confirmed that Stimpson’s original description aligned well with the specimens and description provided by Bate (d’Udekem d’Acoz 2001). As a result, the name *H.australiensis* was retrieved. Although *H.australiensis* has been recognised as a valid species, its taxonomic records are currently limited to Australia, with no documented occurrences in other locations. Recently, two *Hippolyte* specimens were examined from the collections of the Marine Biological Museum, Chinese Academy of Sciences (MBMCAS). These specimens, collected from the South China Sea in 1987, were identified as *H.australiensis* after detailed examination and comparison with known *Hippolyte* species; thus, representing the first record of this species from Chinese waters. This finding significantly expands the known distribution range of *H.australiensis* from the South Pacific to include the North Pacific.

## Materials and methods

Specimens were captured from Daya Bay, South China Sea, by snorkelling in a seaweed bed using a hand-held net in 1987. The specimens were preserved in 75% ethanol and stored at the Marine Biological Museum, Chinese Academy of Sciences. Morphological examination and illustration of the preserved specimens were conducted using a stereomicroscope (Nikon SMZ 1500, Japan). The postorbital carapace length of each specimen was measured to the nearest 0.1 mm using a vernier caliper. The length ratios of various body parts were calculated following the method proposed by d’Udekem d’Acoz (1996). The following abbreviations were used: St. for sampling station and CL for postorbital carapace length.

## Taxon treatments

### Hippolyte australiensis

(Stimpson, 1860)

8AB0168E-56C3-5CCA-9A41-69FC66881765

https://www.marinespecies.org/aphia.php?p=taxdetails&id=515274

*Virbiusaustraliensis*: Stimpson (1860), p. 35 (type locality: Sydney Harbour, Australia); Haswell (1882), p. 186.
*Caradinacincinnuli*: Bate (1863) p.500, pl. 40, fig. 3 (type locality: Gulf St Vincent, Australia); Haswell (1882), p. 183.
*Hippolyteventricose*: Holthuis (1947), p. 16, p.55; Chace (1997), p. 49.
*Hippolyteaustraliensis*: Kemp (1914), p. 98, pl. 2, fig. 6; Hale (1928), p. 91, fig. 19; Edgar (1997), p. 192; Debelius (1999), p. 132; d’Udekem d’Acoz (2001), p. 37, fig. 1-5 (re-description).

#### Materials

- **Type status:** Other material. **Occurrence:** individualCount: 2; sex: 1 male, 1 female; lifeStage: adult male CL 3.8 mm, adult female CL 3.9; reproductiveCondition: non-reproductive; disposition: in collection; occurrenceID: CD8724B4-CD14-55DE-8840-7D8CC59CD738; **Taxon:** scientificName: *Hippolyteaustraliensis* (Stimpson, 1860); kingdom: Animalia; phylum: Arthropoda; class: Malacostraca ; order: Decapoda; family: Hippolytidae; genus: Hippolyte; specificEpithet: australiensis; taxonRank: species; scientificNameAuthorship: (Stimpson, 1860); taxonomicStatus: accepted; **Location:** higherGeography: the northwestern Pacific; continent: Asia; waterBody: South China Sea; country: China; locality: Daya Bay; minimumDepthInMeters: 3; maximumDepthInMeters: 5; verbatimLatitude: 22.5651°N; verbatimLongitude: 114.6633°E; **Identification:** identifiedBy: Zhibin Gan; dateIdentified: 1 October 2023; identificationReferences: d’ Udekem d’ Acoz 2001; **Event:** samplingProtocol: handheld net; samplingEffort: snorkeling in a seaweed bed; eventDate: 1 March 1987; habitat: seaweed bed; fieldNumber: St. F11-7-29; **Record Level:** type: Event; language: English; institutionID: Marine Biological Museum of the Chinese, Academy of Sciences (MBMCAS); collectionID: MBM129577

#### Description

**Figure 1.**
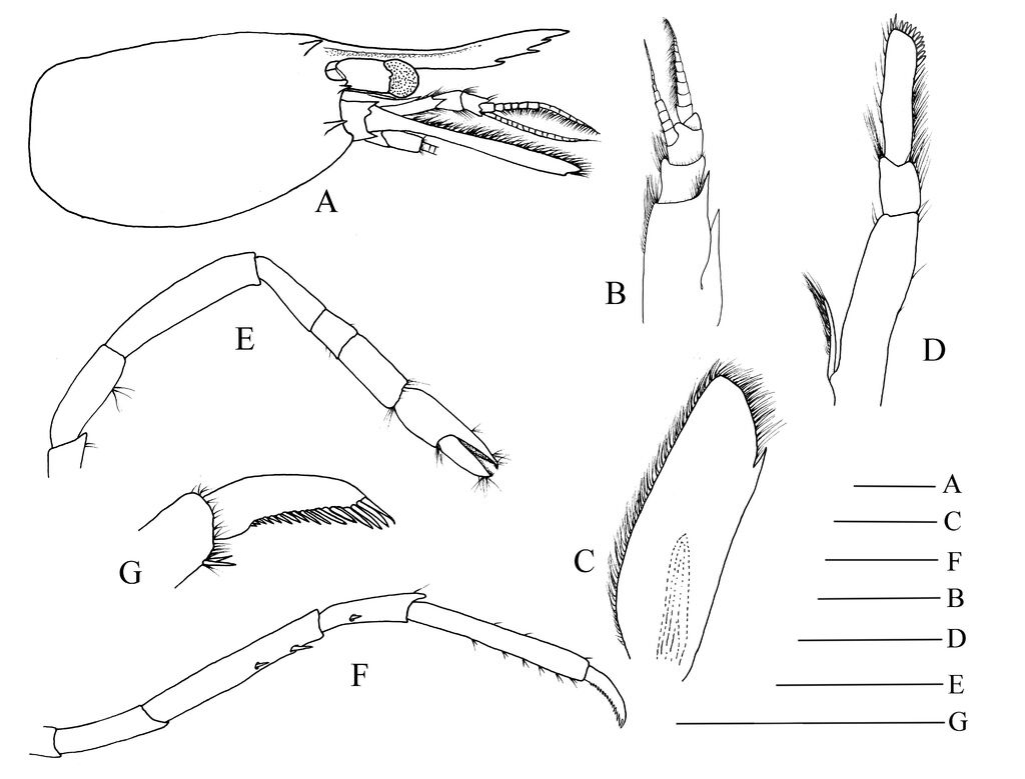
*Hippolyteaustraliensis* (Stimpson, 1860), MBM129577, male. **A** carapace and appendages, lateral; **B** antennular, dorsal; **C** scaphocerite, dorsal; **D** third maxilliped, lateral; **E** second pereiopod, lateral; **F** third pereiopod, lateral; **G** dactylus of the third pereiopod, lateral. Scales: 1.0 mm.

Outline fairly robust, glabrous, without morphological sexual dimorphism. Ratio lateral length/height of carapace 1.65–1.78 (Fig. 1A). Rostrum 7.10–7.25 times as long as high, slightly shorter than carapace, distinctly overreaching antennular peduncle, nearly reaching to end of scaphocerite, proximal part depressed, with long and distinct lateral carina, dorsal border unarmed, distal 0.47–0.56 of ventral border with three teeth. Antennal spine overreaching inferior orbital angle. Hepatic spine slightly overreaching anterior edge of carapace. Pterygostomian angle protruding (Fig. 1A).

Eye well-developed, nearly reaching to stylocerite apex; cornea semi-spherical, without ocellus, shorter and broader than stalk (Fig. 1A). Antennular peduncle reaching to mid-length of scaphocerite, first segment of antennular peduncle with one well-developed distolateral tooth, inner ventral tooth on 0.63 of first segment (excluding distolateral tooth); stylocerite robust, reaching to 0.72–0.75 (distolateral tooth included) or 0.89–0.92 (distolateral tooth excluded) of first segment; second segment of antennular peduncle 0.87–0.89 times as long as broad in dorsal view, approximately 1.26–1.33 times as long as third segment in dorsal view; outer antennular flagellum shorter than inner, flagella with proximal 5–7 segments thicker than distal ones (Fig. 1B). Scaphocerite 3.01–3.11 times as long as wide, distolateral spine of scaphocerite far from reaching distal margin of blade, distolateral spine and blade separated by a notch (Fig. 1C).

Mouthparts with morphology typical of the genus *Hippolyte*. Third maxilliped reaching to 0.22–0.31 of scaphocerite when extended forward; exopod relatively short, about reaching to mid-length of antepenultimate segment of endopod; ultimate segment (excluding apical spine) of endopod 1.82–2.01 times as long as penultimate segment, distal third armed with 9–12 strong spines; antepenultimate segment nearly equal length to the last two segments combined (Fig. 1D).

First pereiopod short, but robust, reaching to end of basicerite when extended forward, tip of fixed finger with three massive spines, tip of dactylus with four massive spines. Second pereiopod slightly overreaching distolateral spine of scaphocerite when extended forward, carpus with three subsegments, first subsegment 1.83–2.09 times as long as second subsegment, third subsegment subequal in length to first subsegment; first subsegment 2.58–2.76 times as long as wide, second subsegment 1.18–1.26 times as long as wide, third subsegment 2.13–2.35 times as long as wide. Cutting edges of chela not denticulate, outer margin of fingers with long simple setae, tip of fixed finger and dactylus armed with three spines, respectively (Fig. 1E). Third to fifth pereiopods similar in shape, but slightly decreasing in size. Third pereiopod (Fig. 1F) nearly reaching to distal end of scaphocerite when extended forward; inner border of dactylus armed with 14–16 spines, with distal four spines largest (Fig. 1G); propodus 6.53–6.98 times as long as wide, armed with 6–7 pairs of spines on ventral margin; carpus 2.89–3.12 times as long as wide, armed with one lateral spine; merus 6.38–6.54 times as long as wide, armed with 2–3 lateral spines. Merus of fourth and fifth pereiopod armed with 1–2 lateral spines.

Third pleomere geniculately curved. Ratio dorsal length/height of the sixth abdominal segment 1.86–1.97. First pleopod of male with appendix masculine shorter than appendix interna, furnished with five apical setae. Telson longer than sixth pleomere, posterior margin armed with four pairs of spines, without intermediate spinule or seta; dorsal surface armed with two pairs of spines situated on distal 0.35–0.41 and 0.59–0.62 telson length, respectively.

##### Distribution

**Figure 2.**
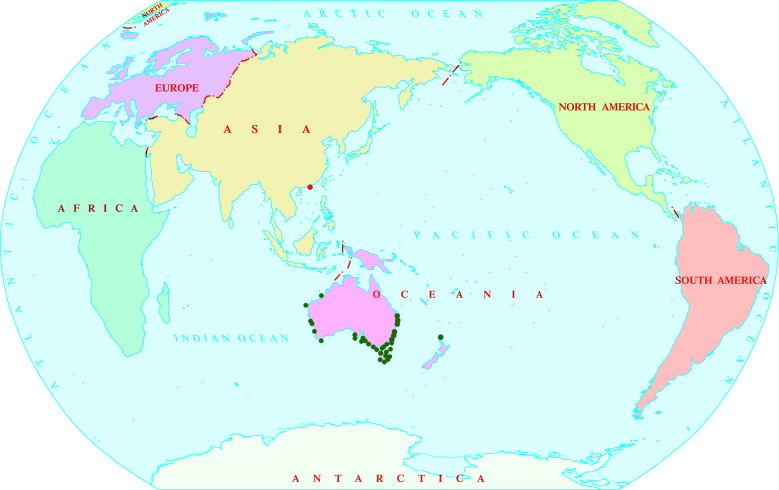
Geographic distribution of *Hippolyteaustraliensis* (Stimpson, 1860). Red dot indicates the newly-recorded location.

The species was previously known to inhabit the western, southern and eastern coastlines of Australia (d’Udekem d’Acoz 2001) and also recorded by GBIF (https://www.gbif.org/species/5799551) and OBIS (https://obis.org/taxon/515274) from the northern coastline of New Zealand. Our present study reports the occurrence of the species for the first time in the South China Sea, within the north-western Pacific region (Fig. 2).

## Discussion

The biodiversity of the genus *Hippolyte* is quite high, both in morphology and genetics (d’Udekem d’Acoz 1996, Terossi and Mantelatto 2012, Anastasiadou et al. 2022). However, the *Hippolyte* species diversity is often underestimated, particularly for the “*H.ventricosa* H. Milne Edwards, 1837” species complex (De Grave et al. 2014, Terossi et al. 2017, Gan and Li 2019). *Hippolyteaustraliensis*, a member of this species complex, was considered as a junior synonym of *H.ventricosa* for an extended period (Holthuis 1947, Chace 1997). It was only after d’Udekem d’Acoz (2001) re-described and retrieved this species that *H.australiensis* was re-established as a valid species. Since then, there have been no taxonomic reports on *H.australiensis*. The present study has identified specimens that correspond closely to the re-description and illustrations of d’Udekem d’Acoz (2001), especially for the diagnostic characters, such as the presence of a long, distinct lateral carina on the rostrum, the dorsally unarmed rostral border, five apical setae on appendix masculine and four prominent terminal spines on the dactylus of the last three pereiopods. Our finding of *H.australiensis* occurring in the South China Sea not only further confirms the judgement of d’Udekem d’Acoz (1999) and d’Udekem d’Acoz (2001), but also greatly extends the geographic distribution of this species from the South Pacific to the North Pacific.

Additionally, we also observed minor morphological differences between the present specimens and the Australian specimens. Specifically, the present specimens have 14–16 spines on the inner border of the dactylus of the third pereiopod (including the four terminal spines), while that of the Australian specimens have 10–13; the third maxilliped of our specimens is slightly longer than that of the Australian specimens in proportion, reaching to 0.22–0.31 of the scaphocerite, as opposed to 0.20 of the scaphocerite in the Australian specimens. However, these morphological variations fall within the range of intraspecific variation observed in *Hippolyte* species throughout their geographic populations.

## Supplementary Material

XML Treatment for Hippolyte australiensis
